# Immuno-protective impact and clinical translation of radioprotective agents in cancer radiotherapy

**DOI:** 10.3389/fimmu.2025.1610296

**Published:** 2025-07-04

**Authors:** Yiyong Huang, Xiaolan Lv, Tao Si, Xia Meng, Xiaolin Liao, Pengfei Zhang, Zheng Peng, Zheyi Zhou, Ping Yi, Shigao Huang

**Affiliations:** ^1^ Department of Clinical Laboratory, Liuzhou Traditional Chinese Medical Hospital, Liuzhou, Guangxi, China; ^2^ Department of Clinical Laboratory, Liuzhou Maternity and Child Healthcare Hospital, Liuzhou, Guangxi, China; ^3^ Department of Clinical Oncology, Liuzhou Traditional Chinese Medical Hospital, Liuzhou, Guangxi, China; ^4^ Department of Orthopedics, Liuzhou Traditional Chinese Medical Hospital, Liuzhou, Guangxi, China; ^5^ Department of Pulmonary and Critical Care Medicine, Liuzhou Traditional Chinese Medical Hospital, Liuzhou, Guangxi, China; ^6^ Postdoctoral Research Workstation, Wuzhou Red Cross Hospital, Wuzhou, Guangxi, China; ^7^ Department of Neurology, Liuzhou Traditional Chinese Medical Hospital, Liuzhou, Guangxi, China; ^8^ Department of Gastroenterology, Liuzhou Traditional Chinese Medical Hospital, Liuzhou, Guangxi, China; ^9^ Department of Radiation Oncology, Xijing Hospital, Fourth Military Medical University, Xi’an, China

**Keywords:** radioprotector, protective mechanism, immuno-protective, clinical application, cancer radiotherapy

## Abstract

Radiotherapy, as a key component of the comprehensive treatment system for malignant tumors, not only facilitates precise tumor destruction but also necessitates the strategic use of radioprotective agents to regulate immune responses and mitigate toxicity in normal tissues. Revealing the molecular biological mechanisms of ionizing radiation damage, such as DNA double-strand breaks, oxidative stress responses, and abnormal cell cycle regulation is critical for the development of clinically effective radioprotective drugs. Such advancements hold dual significance in enhancing patient outcomes and improving clinical efficacy. This paper explores the classification of radioprotective agents, and their diverse mechanisms of action, including free radical scavenging, regulation of redox enzyme systems, suppression of ionizing radiation-induced inflammation, and apoptosis-related immune damage. And, it also examines the challenges and prospects of their clinical translation. This study aims to provide important theoretical framework for the development of radioprotective agents to contribute to future advancements in radiation therapy.

## 1 Introduction

**Figure 1:**
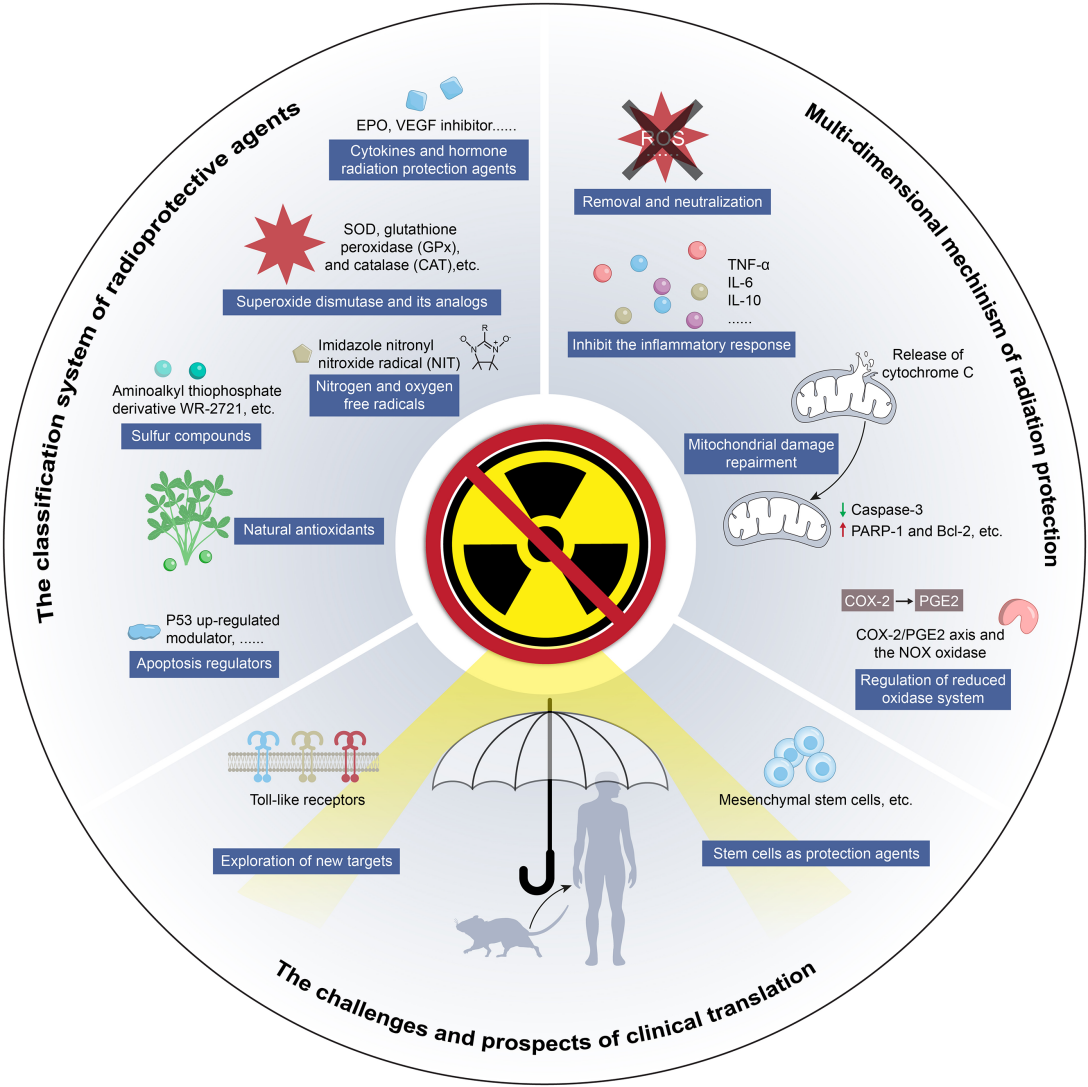
Schematic of study design from classification of radiation protection agents to challenge and prospects of clinical translation.

Radiation therapy is a fundamental component of comprehensive cancer treatment and plays a crucial role in clinical oncology (1, 2). Epidemiological studies indicate that approximately 70% of patients with malignant tumors require radiation therapy during their treatment course (3, 4). However, while ionizing radiation effectively eliminates tumor cells, it also inevitably damages adjacent normal tissues, with dose-limiting toxicity posing a major challenge to its clinical application (5). To mitigate this issue, the development and application of radioprotective agents have emerged as a key strategy for enhancing the safety of radiation therapy. Radioprotective agents encompass various types with complex mechanisms of action, and most remain in preclinical research stages (6–8). Currently, only two radioprotective drugs are approved by the U.S. Food and Drug Administration (FDA) for clinical use: amifostine (9, 10), which scavenges free radicals and selectively protects normal tissues, and palifermin (11), a recombinant human keratinocyte growth factor (KGF) that promotes epithelial cell repair. Despite this, numerous potential radioprotective agents are under investigation, including free radical scavengers, superoxide dismutase (SOD) and its analogs, nitric oxide compounds, natural antioxidants, cytokines, and hormone-like substances that regulate apoptosis. This article aims to summarize the types of radioprotective agents and their mechanisms of action. These mechanisms include directly scavenging reactive oxygen species (ROS) generated by radiation, neutralizing free radical toxicity; reducing oxidative stress damage, inhibiting inflammatory responses, maintaining cellular homeostasis, blocking abnormal apoptosis pathways and activating DNA damage repair systems. Additionally, this review explores the challenges and future prospects of clinical applications, with the goal of contributing to the optimization of radiation therapy efficacy (**Figure 1**).

## 2 Classification of radiation protection agents

### 2.1 Sulfur compounds

Sulfur compounds represent a pioneering category of radiation protection agents, playing a pivotal role in advancing the understanding of their mechanisms in modern radiation protection research (12–14). The unique thiol (−SH) groups in these compound molecules endow them with distinctive free radical scavenging capabilities. By donating hydrogen, these compounds effectively neutralize ROS generated by radiation, thereby interrupting the free radical chain reaction. The earliest sulfur-containing compound with documented radioprotective efficacy dates back to World War II, when researchers observed that administering supra-physiological doses of cysteine significantly improved the survival rates of mice following whole-body irradiation (15, 16). Subsequent studies confirmed that mercaptoethylamine, a metabolite of cysteine, also exhibited protective effects in irradiated animals. However, these early compounds were associated with significant neurotoxicity and gastrointestinal side effects at effective protective doses, severely limiting their clinical translation. After systematically screening over 4,400 compounds, researchers identified WR-2721 (17),a phosphorylated aminothiol compound (amifostine), which demonstrated excellent radioprotective efficacy with relatively manageable toxicity.

After structural optimization, this drug demonstrated unique tissue selectivity, effectively targeting and neutralizing free radicals. Its multiple mechanisms of action, including metal ions chelation, play a crucial role in radioprotection. Due to differences in microenvironment acidity and enzyme activity, tumor tissues struggle to activate the drugs, thus maintaining radiotherapy sensitivity. Extensive clinical evidence led to the FDA’s approval of amifostine in 1996 as the only drug with dual indications for both cytoprotection and radioprotection. Studies have shown that it significantly reduces the incidence and severity of radiation-related oral mucositis in patients with head and neck cancer without compromising local tumor control rate (17). This breakthrough not only validates the clinical value of sulfur-containing compounds but also establishes an important paradigm for the development of novel targeted radioprotective agents.

Amifostine, a broad-spectrum cytoprotective agent, exerts its protective effects through three primary mechanisms: directly scavenging oxygen free radicals generated by ionizing radiation via its thiol groups; enhancing the activity of DNA repair enzymes to facilitate damage repair; and inducing the formation of functional hypoxic microenvironment in normal tissues (18). Additionally, amifostine exhibits significant selective protection differences between normal and tumor tissues. Clinical studies have confirmed that it can reduce radiation-induced damage in normal tissues by 60%-80% while providing minimal protection on tumor tissues. This selective effect is primarily attributed to the high expression of alkaline phosphatase in normal tissues, which efficiently catalyzes drug activation; the abnormal vascular distribution characteristic of tumor tissues, which restricts drug penetration; and the inhibitory effect of the acidic tumor microenvironment on drug metabolism (18). Despite its efficacy, the clinical application of amifostine has several limitations: (1) time window limitation – the optimal dosing window is 20–30 minutes before radiotherapy, beyond which its protective efficacy declines significantly; (2) limited administration routes – currently, only intravenous administration is available, with no oral formulations developed; (3) safety concerns – approximately 30%-60% patients experience acute hypotension, persistent nausea, vomiting, and other treatment-related adverse effects, which can lead to treatment interruption in severe cases. To overcome these limitations, the development of new sulfur-containing radiation protection agents should focus on breaking through three major challenges: optimizing drug metabolism and pharmacokinetics, developing multi-pathway drug delivery systems, and minimizing systemic toxicity.

### 2.2 Cytokines and hormone radiation protection agents

Cytokines, including interleukin-1 (IL-1), tumor necrosis factor-alpha (TNF-α), and granulocyte colony-stimulating factor (G-CSF), activate differentiation pathways in bone marrow hematopoietic stem and progenitor cells. Administering these cytokines within a 24-hour treatment window before and after radiation can reduce the risk of radiation damage (19, 20). However, their clinical application is limited by the systemic inflammatory response they trigger. Erythropoietin (EPO), although effective in correcting radiation anemia, may promote tumor angiogenesis through the vascular endothelial growth factor (VEGF) signaling pathway. A clinical trial in head and neck cancer patients (21) showed that EPO administration led to tumor growth and did not improve overall survival or progression-free survival. Conversely, bevacizumab, a VEGF inhibitor, demonstrated potential in a small-scale clinical trial involving brain tumor patients by reducing radiation-induced tissue necrosis (22).

Certain fibroblast growth factor (FGF) family members also exhibit radioprotective properties. Notably, recombinant human FGF-7, also known as recombinant human KGF or palifermin. However, the large-scale production of recombinant FGF is challenging and costly, highlighting the urgent need for FGF peptide analogs. KGF interacts with its receptor on epithelial cells to activate signaling pathways that promote epithelial cell proliferation, migration, and differentiation, enhance DNA repair, and counteract ROS (23). Palifermin is the first FDA-approved drug for preventing severe oral mucositis in hematopoietic stem cell transplant patients. Preclinical studies have shown that palifermin can improve oral mucositis in rats exposed to high levels of radiation, protect parotid gland function, and reduce lung damage (6, 24). Although palifermin carries a theoretical risk of stimulating tumor growth, no clinical evidence has yet confirmed this risk, and research is ongoing (25). Additionally, clinical studies have demonstrated that palifermin mitigates the severity and duration of oral mucositis in patients undergoing radiotherapy and chemotherapy for hematologic malignancies (26, 27).

Recombinant human prolactin (rhPRL) and growth hormone (GH) jointly regulate the development of the human hematopoietic system, with PRL playing a crucial role in erythropoiesis. rhPRL promotes red blood cell (RBC) regeneration, and both *in vitro* and *in vivo* studies have confirmed its involvement in the development and maturation of the hematopoietic and immune systems (28). The radioprotective mechanism of PRL facilitates hematopoietic regeneration and/or enhances the hematopoietic system’s tolerance to radiation exposure (29).

### 2.3 Nitrogen and oxygen free radicals

Nitrogen-oxygen free radicals, as stable free radical compounds with unique electronic structures, hold significant potential in radiation protection research. Their protective mechanisms primarily involve two pathways: one is through single-electron transfer (SET) to directly neutralize ROS, and the other mimics the function of SOD to catalyze the disproportionation reaction of superoxide anions. Among nitrogen-oxygen free radical derivatives, tetramethylpiperidine nitrogen-oxygen free radical (Tempol) is the most extensively studied. Studies have shown that Tempol not only effectively reduces radiation-induced cytotoxicity in mammalian cell models but also provides significant protection in whole-body irradiated mice without compromising tumor radiosensitivity (30–32). However, this compound can easily cause dose-limiting toxicity at effective protective doses, including systemic hypotension, reflex tachycardia, and abnormal central nervous system excitability (32). To break through the limitations of traditional nitrogen-oxygen free radicals, Huang, et al. (5, 8). synthesized a novel imidazoline nitrogen oxide compound, triphenylphosphine nitronyl nitroxide (TPP-NITs). Mechanism studies indicate that these compounds exert their effects through multiple pathways, including scavenging free radicals to reduce oxidative stress, modulating apoptosis pathways, and antagonizing inflammatory damage to protect the spleen, marking a technological breakthrough in subcellular-targeted protection. The nitrogen oxide radical module is responsible for ROS scavenging, while the triphenylphosphine (TPP) moiety acts as a mitochondrial localization signal, directing the compound to the inner mitochondrial membrane and significantly enhancing its protective effects on radiation-sensitive organelles (5) This targeted delivery strategy not only improves protective efficiency but also provides new insights into addressing the issue of tissue selectivity limitations in traditional protective agents. However, further studies are required to evaluate its long-term toxicity and clinical applicability.

### 2.4 Natural antioxidants

Natural antioxidants, including vitamins and plant extracts, possess oxygen-free radical-scavenging properties, making them potential candidates for radiation protection. Vitamin C and E have been shown to reduce chromosomal damage, mutations, and apoptosis in mammalian cells caused by radiation. Vitamin A and β-carotene enhance the tolerance of mice to high doses of radiation, while glutathione (GSH) and coenzyme Q10 mitigate radiation-induced oxidative stress (33, 34). The primary advantage of these natural antioxidants is their minimal toxicity; however, their radioprotective efficacy is lower than that of synthetic drugs such as amifostine. Additionally, their non-selective free radical-scavenging properties result in the protection of both tumor and normal tissues. In animal models, melatonin not only protects normal tissues from radiation damage but also exhibits anti-tumor effects. As both a radiosensitizer and a radioprotector, melatonin has entered Phase I clinical trials, showing good drug tolerance (35). However, the clinical trial failed to demonstrate a significant extension of survival in the treated group compared to the control group.

Genistein is a non-specific protein kinase inhibitor that can clear ROS in the body and reduce nuclear factor kappa B (NF-κB) activity, thereby modulating the expression of cytokines, chemokines, immune receptors, and adhesion molecules (7). Mahmood et al. found that genistein improves respiratory rate in animals exposed to lung irradiation and delays their death time (36, 37). However, its limitation lies in rapid elimination within the body and a narrow therapeutic window. Siwu Decoction is a traditional Chinese herbal decoction composed of Rehmannia glutinosa, Angelica sinensis, Paeonia lactiflora, and Ligusticum chuanxiong. It can promote the recovery of peripheral blood cells in mice subjected to whole-body irradiation. It also enhances hematopoietic progenitor cell colony formation (38–41).

### 2.5 Superoxide dismutase and its analogs

The maintenance of cellular redox homeostasis relies on core antioxidant enzyme systems such as SOD, glutathione peroxidase (GPX), and catalase (CAT). The SOD family, comprising Cu/Zn-SOD, Mn-SOD, and extracellular SOD subtypes, plays a crucial role in protecting against radiation by specifically catalyzing the disproportionation of superoxide anions in the cytoplasm, mitochondria, and extracellular space (42). Although animal experiments have shown that SOD effectively scavenges radiation-induced ROS and mitigates chronic oxidative stress, its clinical translation still faces challenges. Current research primarily follows two strategies: developing new SOD analogs by designing small molecules to circumvent the structural limitations of natural enzymes and employing gene therapy techniques using non-viral vectors, such as plasmid-liposome (PL) complexes, for targeted delivery. Studies have demonstrated that Mn-SOD-PL gene therapy selectively protects normal tissues, such as the lungs and esophagus, in lung cancer models, without affecting tumor radiosensitivity (43). However, the clinical translation of this technology still requires systematic evaluation of its pharmacokinetic and potential toxicity. The development of SOD mimics has emerged as a key focus in radiation protection research. These small molecule compounds containing metal active centers such as manganese (Mn) and copper (Cu), have notable advantages over natural SOD (44), including an extended half-life, an expanded therapeutic window, and reduced immunogenicity (45). Current research hotspots include the development of bifunctional mimics, such as the EUK series compounds, which possess both SOD and CAT activities, showing synergistic radioprotective effects in organs like the lungs and kidneys (46).

### 2.6 Apoptosis regulators

The p53 upregulates apoptosis regulators, such as p53 up-regulated modulator of apoptosis (PUMA), which serve as key initiators of the mitochondrial apoptosis pathway and play a central role in the radiation-induced apoptosis cascade in normal tissue cells. Studies have shown that PUMA gene knockout mice exhibit extended survival after 10 Gy whole-body irradiation compared to wild-type mice, with a reduced apoptosis rate in hematopoietic stem cells. Given that over half of human malignant tumors show p53 pathway inactivation, small molecule inhibitors targeting PUMA demonstrate significant tissue selectivity *in vitro* (47). These inhibitors enhance radiation resistance in normal epithelial cells by 4.5 times, while showing no significant effect on sensitivity to p53 mutant tumor cells. This differential regulatory property provides an important research direction for developing next-generation intelligent radiation protectants.

Entolimod, a derivative of Salmonella flagellin protein, exerts its radiation protection effect by specifically binding to toll-like receptor 5 (TLR5) and activating the NF-κB signaling pathway. Its radiation protection is mainly reflected in: inhibiting p53-mediated apoptosis pathways; and upregulating the expression of anti-apoptotic factors such as Bcl-2 (48). Notably, this drug has specific protective effects on normal tissues expressing TLR5, such as intestinal mucosa and bone marrow hematopoietic system (49). Animal experiments have shown that it can significantly improve hematopoietic function recovery in rhesus monkeys and mice exposed to lethal doses of radiation, and promote the regeneration rate of gastrointestinal mucosa (50). Additionally, entolimod shows significant efficacy in reducing radiation pneumonitis and pulmonary fibrosis (51), and no radiation protection effect was observed on tumor tissues, demonstrating a good therapeutic safety margin.

## 3 Multi-dimensional mechanism of radiation protection

### 3.1 Removal and neutralization of free radicals produced by ionizing radiation

**Figure 2:**
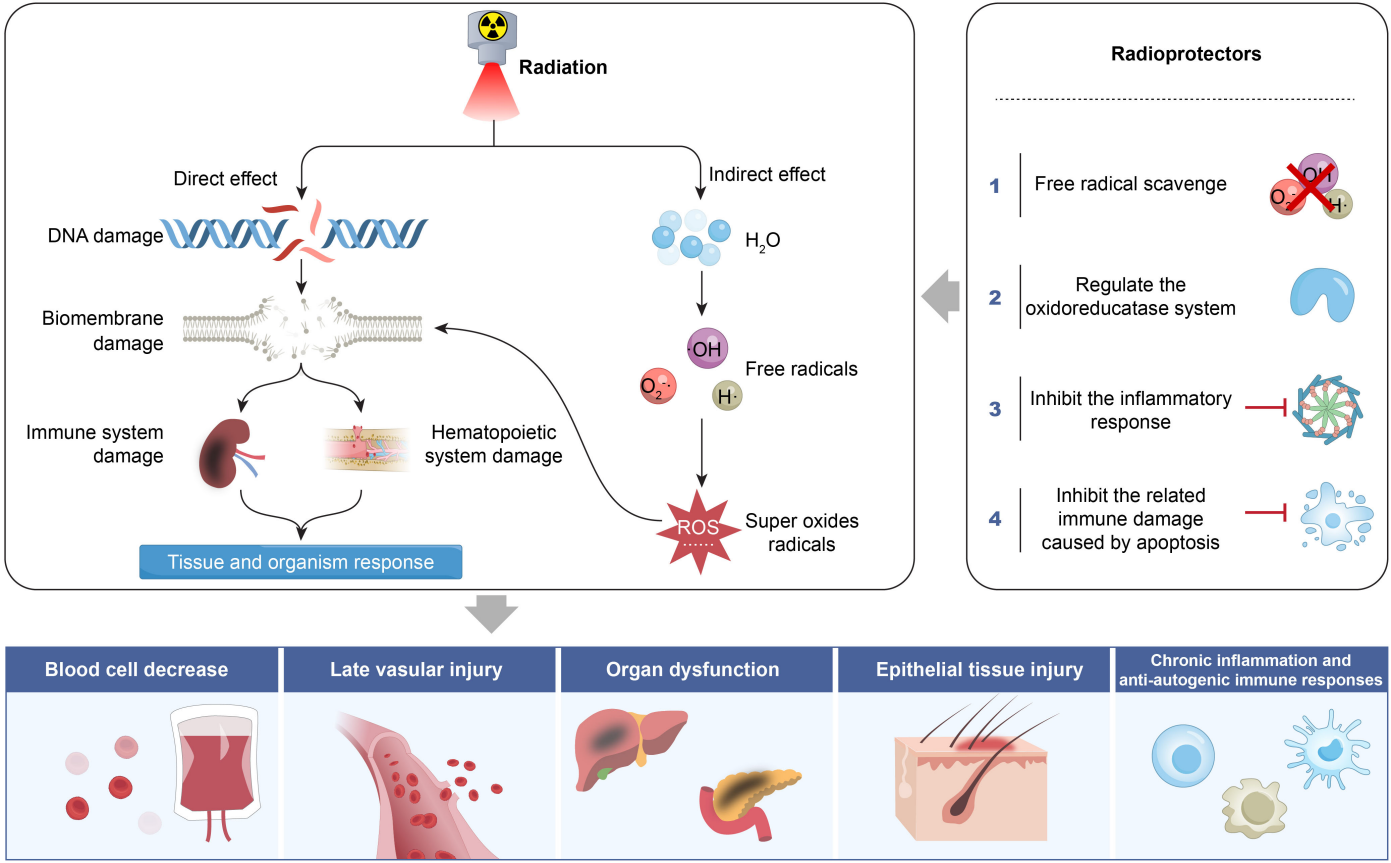
Mechanism of radiation damage to tissue and organism response.

Ionizing radiation includes the formation of highly reactive free radicals (e.g., OH, O_2_^-^·, H·) through water radiolysis. These free radicals cause multi-target damage, including lipid peroxidation of the cell membrane’s phospholipid bilayer, oxidation of protein thiol groups, and DNA double-strand breaks, as indicated by increased γ-H2AX foci. Free radicals that escape neutralization by endogenous antioxidant systems. CAT compete for electrons from surrounding cells, blood vessels, proteins, lipids, and DNA, thereby damaging normal tissues, thereby damaging normal tissues. Consequently, scavenging and neutralizing free radicals is a critical strategy to mitigate ionizing radiation-induced tissue damage (**Figure 2**). The mechanisms for free radical scavenging typically fall into two categories: hydrogen atom transfer (HAT) and SET (52). Amifostine, a thiol-based compound approved by the FDA for cellular radiation protection, donates hydrogen atoms through its thiol groups to neutralize free radicals and facilitates DNA repair. Similarly, melatonin not only scavenges various oxygen free radicals and protects DNA, but also enhances some protection against antioxidant enzymes such as CAT, GPX, and SOD, indicating its role in reducing radiative damage by scavenging free radicals (53, 54). Additionally, studies have confirmed that GSH, another thiol-containing antioxidant, effectively scavenges free radicals and improves the cognitive abilities of radiation-exposed mice (55). Natural antioxidants reduce the effects of ionizing radiation through antioxidant, free radical scavenging, and anti-inflammatory mechanisms (56, 57). The nanomedicine (NPs-TPP-NIT) developed by Huang et al. (8). significantly prolongs circulation time without noticeable toxicity to mice and cells. It effectively protects L-02 cells from X-ray-induced radiation damage by enhancing mitochondrial membrane potential and inhibiting apoptosis. Moreover, it notably improves survival outcomes in irradiated mice by extending survival time and increasing survival rates. The treatment promotes the recovery of peripheral blood and bone marrow profiles in mice, enhances endogenous splenic colony formation ability, significantly reduces oxidative stress damage to the spleen of irradiated mice, and inhibits both apoptosis and inflammatory damage (5).

### 3.2 Regulation of reduced oxidase system to reduce oxidative stress injury

Radiation therapy induces programmed cell death, which subsequently triggers an inflammatory storm, leading to the release of key mediators such as interleukin-1 beta (IL-1β), TNF-α, and transforming growth factor-beta (TGF-β) (58). These mediators activate the cyclooxygenase-2/prostaglandin E2 (COX-2/PGE2) signaling axis and the NADPH oxidase (NOX) system, leading to increased superoxide anion production and thereby exacerbating oxidative stress-induced damage. The activation of COX-2 and NOX system contributes to persistent oxidative stress, genomic instability the generation of chronic free radicals (59), thereby aggravating radiation-induced pneumonitis, tissue fibrosis, and related vascular injuries (**Figure 2**). The NOX system includes NOX1–5 and DUOX1-2, both of which play a role in chronic oxidative stress and fibrosis. Multiple studies have shown that NOX inhibitors, such as vanillin acetone and diphenylhydantoin ammonium (DPI), suppress NOX1, NOX2, and NOX3, while metformin and resveratrol can inhibit NOX4 and NOX5 (60–62). Additionally, celecoxib can inhibit COX-2, thereby reducing the effects of ROS and nitric oxide synthase (NOS), alleviating radiation-induced inflammation and fibrosis in organs such as the lungs, intestines, heart, and salivary glands (63, 64).

Cells contain redox-sensitive genes, whose promoters harbor redox-regulated sensor elements. Certain compounds enhance radiation protection by upregulating antioxidant enzyme expression, primarily through activation of the nuclear factor erythroid 2-related factor 2 (Nrf2)/antioxidant response element (ARE) pathway (65, 66). The NF-κB transcription factor is a redox-sensitive protein that directly or indirectly regulates the expression of many genes, with its expression upregulated in response to oxidative stress. Therefore, activating the NF-κB pathway after radiation exposure is considered a potential radiation protection mechanism (52). Huang et al. also found that preventing the activation of the NF-κB pathway can confer protective effects on immune organs (5).

### 3.3 Inhibit the immune damage caused by apoptosis and reduce mitochondrial damage

Mitochondrial pathways are involved in apoptosis through several mechanism (67). This process rapidly activates caspase-9, caspase-3, and caspase-8, ultimately triggering apoptosis. Then, the Bcl-2 family can bind to Bax, causing pores in the outer mitochondrial membrane, and accelerating the release of Cyt c (68). Finally, p53-initiated cell cycle arrest contributes to apoptotic signaling. Ionizing radiation induces mitochondria ROS, promoting depolarization of the mitochondrial membrane potential and the release of Cyt c, leading to apoptosis (69). Small molecules that inhibit apoptosis without compromising DNA repair can serve as valuable radiation protectants. In the Bcl-2/Caspase-3/Polymerase-1 (PARP-1) apoptosis signaling pathway, flavonoids extracted from Aspergillus flavus (FRT) inhibit apoptosis by downregulating Caspase-3 and upregulating PARP-1 and Bcl-2 protoforms, thereby achieving radiation protection (70). Some dual-action drugs protection for acute radiation syndrome (ARS) and radiation-wound injuries via regulating Bcl-2 and caspases in the apoptotic process (71). In the V79 cell model, pre-treated with isoflavones 24 hours before exposure to 8Gy^60^Coγ-rays not only eliminates ROS but also stops the cell cycle in the G2 phase and inhibits apoptosis, showing as a potential radioprotective agent (70).

### 3.4 Inhibit the inflammatory response caused by ionizing radiation

During radiation therapy, damaged cells produce large amounts of inflammatory mediators, which trigger a series of immune responses under the influence of macrophages and lymphocytes, exacerbating the damage caused by radiation. However, certain inflammatory factors also facilitate cell repair. Several radioprotective agents mitigate radiation injury by accelerating cell repair, including hormones or steroid analogs, such as estrogens and androgens (72); and interleukins, TNF, hematopoietic growth factors (G-CSF, GM-CSF, M-CSF, IL-3), interferons, and immunomodulatory peptides (73). These agents primarily function by activating of NF-κB pathway. NF-κB is a “rapid-acting” primary transcription factor that is inactive in the cytoplasm. It can be activated by IL-1β, TNF, lipopolysaccharide receptors, bacterial and viral antigens, ROS, and ionizing radiation. It exhibits a bidirectional effect, both promoting tumor cell growth and mediating normal immune responses (74, 75). Activating NF-κB synergizes with the activator protein-1 (AP-1)/signal transducer and activator of transcription 3 (STAT-3) signaling pathway to promote the expression of cell proliferation genes and inhibit the expression of stem cell apoptosis genes, enhancing cell repair (76, 77). These drugs are used for emergency treatment and early intervention in ARS. It is of great significance.NF-κB can be activated by G-protein receptor mediators’ amines, nucleosides, prostaglandins, and angiotensin as well as hormone nuclear receptors, which have obvious protection against radiation-induced bone marrow injury (78).

## 4 Challenges and prospects of radiation therapy protection

### 4.1 Exploration of new targets and improvement of radiation protection capability

Targeted toll-like receptors (TLRs): TLRs are expressed in various mammalian cells and can activate and upregulate the NF-κB signaling pathway, thereby enhancing cellular radiation resistance. The multiple TLRs subtypes often exhibiting synergistic effects in radiation-induced damage (79, 80). The TLR2 ligand, bacterial lipoprotein, inhibits oxidative reactions by upregulating anti-apoptotic factors and cytokines (81, 82); TLR9, primarily localized in lysosomes, is upregulated in human keratinocytes upon ultraviolet exposure, thereby enhancing cellular radiation resistance. Inactivated Salmonella typhi can inhibit apoptosis, reduce DNA damage, and provide protection to sensitive tissues such as the spleen, bone marrow, and testes because inactivated Salmonella typhi possesses both TLR4 and TLR2 activity and achieves radiation protection by inducing NF-κB p65 subunit translocation (83). The synergistic interaction of TLRs may amplify the protective effect of radiation, leading to the activation of TLRs may produce a wider and more powerful radiation protection effect (84). Targeted miRNA 21: Ionizing radiation can induce microRNAs (miRNAs) expression associated with ROS production, such as let-7 family, miR-15b, miR-21, miR-128, and miR-636. Selective inhibitors can suppress TGF-β R1, thereby eliminating miR-21 and oxidative stress response in bystander cells (85–88). TGF-β upregulates miR-21 expression, and its knockout or inhibition may exert protective or sensitizing effects on radiotherapy (89, 90).

### 4.2 Stem cells as radiation protection agents

Stem cell therapy, as a focal point in the field of radiation protection, has been confirmed by multiple experimental studies to have significant therapeutic and preventive effects on the side effects of radiotherapy. Mesenchymal stem cells (MSCs), as a type of multipotent stem cell, have been shown to effectively mitigate acute radiation-induced lung injury due to their resistance to hypoxia and the overexpression of the manganese superoxide dismutase (MnSOD) gene in MSCs (91). Additionally, bone marrow MSCs can specifically deliver the RSPO1 gene, thereby improving or even curing radiation-induced intestinal damage (92). In terms of reducing liver damage caused by radiotherapy, the combined use of bone marrow MSCs and nigella sativa oil has also shown positive results (93). Furthermore, MSCs provide radiation protection to the hematopoietic system through the Jagged1-Notch2 signaling pathway (94, 95). As a therapeutic approach applicable to various tissues, stem cell therapy aims to restore the intrinsic cellular function of damaged tissues with fewer side effects.

### 4.3 Clinical challenges and prospects

Despite advancements in radiation therapy that have reduced the toxic side effects of radiation to some extent, normal tissues remain vulnerable to radiation damage. Therefore, the development and use of anti-radiation drugs is particularly crucial (96). Historically, the development of anti-radiation drugs focused on synthesizing compounds with free radical scavenging properties. As understanding of intracellular signaling pathways deepened, research strategies have shifted towards targeting the cascade reactions involved in post-radiation cell repair. Many of these drugs exert their anti-radiation effects by increasing the expression of key signaling pathway factors, inhibiting apoptosis, or modulating the balance between fibrotic and anti-fibrotic regulatory factors (97).

The ideal anti-radiation drugs for radiotherapy should possess the following characteristics: significant radiation protection for normal tissues, with minimal or no effect on tumor tissues, to maintain the clinical relevance of the therapy; low toxicity and compatibility for use in combination with other drugs without adverse interaction; and ease of administration, stability, and a broad therapeutic window. Despite ongoing research efforts on anti-radiation drugs, some effective agents have emerged (98), but no single drug has yet fully met all these criteria (97, 99, 100). Future development of radiation protection drugs should focus on several directions. First, developing radiation protection drug formulations with nano targeting to achieve precise targeting of tumor tissues, thereby reducing toxic reactions. Second, identifying specific signaling pathways that differentiate tumors from normal tissues may lead to the development of drugs that selectively protect normal tissues without affecting tumors (101) and target to enhance the radiosensitivity (102). For the clinical application of radioprotective drugs in therapy, two key factors must be met: the drug must not protect tumors, ensuring that it does not interfere with radiotherapy efficacy (5); and it should exhibit low toxicity and minimal side effects, avoiding severe adverse reactions such as intense vomiting that could lead patients to miss their radiotherapy sessions, resulting in a loss. Currently, many radioprotective drug candidates are in various stages of development and it is anticipated that within the next decade, they will enter clinical practice, benefiting a broader range of patients undergoing therapy.

## 5 Conclusion

Radioprotective agents encompass a broad range of substances, and their mechanisms of action are dynamic, continuous, and involve complex biological processes. Currently, most radioprotective agents are still in the preclinical research phase and have not yet been translated into clinical application. This review summarizes the types of radioprotective agents and their mechanisms of protective action: such as scavenging and neutralizing free radicals, reducing oxidative stress damage, inhibiting inflammatory responses, blocking immune damage caused by cell apoptosis, and facilitating DNA repair, which enhances our understanding of their effects of radioprotective agents from multiple perspectives. Such comprehensive knowledge is crucial for the development of novel clinical drugs and for enhancing the overall efficacy of radiotherapy.
